# Single Molecule Analysis of Functionally Asymmetric G Protein-coupled Receptor (GPCR) Oligomers Reveals Diverse Spatial and Structural Assemblies*^♦^

**DOI:** 10.1074/jbc.M114.622498

**Published:** 2014-12-16

**Authors:** Kim C. Jonas, Francesca Fanelli, Ilpo T. Huhtaniemi, Aylin C. Hanyaloglu

**Affiliations:** From the ‡ Institute of Reproductive and Developmental Biology, Department of Surgery and Cancer, Imperial College London, Du Cane Road, London W12 0NN, United Kingdom,; the § Computational Structural Biology Lab, Department of Life Sciences, University of Modena and Reggio Emilia, via Campi 183-41100 Modena, Italy, and; the ¶ Institute for Biomedicine, Department of Physiology, University of Turku, 20520 Turku, Finland

**Keywords:** Bioluminescence Resonance Energy Transfer (BRET), Cell Signaling, G Protein, G protein-Coupled Receptor (GPCR), Structural Biology, Dimer, Oligomer, Super-resolution Imaging

## Abstract

Formation of G protein-coupled receptors (GPCRs) into dimers and higher order oligomers represents a key mechanism in pleiotropic signaling, yet how individual protomers function within oligomers remains poorly understood. We present a super-resolution imaging approach, resolving single GPCR molecules to ∼8 nm resolution in functional asymmetric dimers and oligomers using dual-color photoactivatable dyes and localization microscopy (PD-PALM). PD-PALM of two functionally defined mutant luteinizing hormone receptors (LHRs), a ligand-binding deficient receptor (LHR^B−^) and a signaling-deficient (LHR^S−^) receptor, which only function via intermolecular cooperation, favored oligomeric over dimeric formation. PD-PALM imaging of trimers and tetramers revealed specific spatial organizations of individual protomers in complexes where the ratiometric composition of LHR^B−^ to LHR^S−^ modulated ligand-induced signal sensitivity. Structural modeling of asymmetric LHR oligomers strongly aligned with PD-PALM-imaged spatial arrangements, identifying multiple possible helix interfaces mediating inter-protomer associations. Our findings reveal that diverse spatial and structural assemblies mediating GPCR oligomerization may acutely fine-tune the cellular signaling profile.

## Teaser

**Background:** GPCRs form complex oligomers whose role in signaling is poorly understood.

**Results:** Super-resolution imaging of functionally asymmetric oligomers reveals diverse functional and structural organizations and the ability to alter signal responses.

**Conclusion:** GPCR oligomers may fine-tune receptor signaling by altering the functional role of individual protomers.

**Significance:** Distinct oligomers could be exploited pharmacologically to improve efficacy, selectivity, and/or specificity.

## Introduction

The molecular organization of G protein-coupled receptors (GPCRs)^3^ into monomers, dimers, and oligomers is emerging as a key mechanism in mediating signal diversity and specificity. Di/oligomerization can impact receptor pharmacology, functional selectivity or ligand bias, G protein-coupling, and receptor trafficking (1). However, a fundamental question that remains is whether the functional role of individual receptor protomers are distinct within a GPCR complex and whether altering the role of a protomer in a di/oligomer may change the overall functional output by modulating signal strength or diversity.

Intermolecular cooperativity, also termed functional complementation or trans-activation, has been used as a tool to study the role of GPCR dimerization on receptor function, whereby co-expression of two distinct nonfunctional mutant GPCRs can “rescue” receptor functionality (2–9). Using the luteinizing hormone receptor (LHR) as a model GPCR, we have employed intermolecular cooperation to demonstrate the physiological relevance of class A GPCR homo-di/oligomerization. Transgenic expression of either ligand binding-deficient (LHR^B−^) or signaling-deficient (LHR^S−^) receptors was nonfunctional and had no effect on the hypogonadal phenotype of male LHR knock-out mice. However, co-expression of these two mutant receptors could rescue LHR-dependent signaling and infertility of these knock-out animals (10). Such “designed” functional asymmetry has also been observed in nonmutated receptor dimers and is thought to underlie the mechanisms of functional selectivity and cooperative allosteric regulation between protomers of di/oligomers (11–13).

Detection of GPCR di/oligomerization at a molecular level has primarily utilized resonance energy transfer techniques (14) and, more recently, total internal reflection microscopy (TIRF-M) combined with post-acquisition extrapolation of intensity data to resolve GPCR molecules (15–17). The recent advent of super-resolution imaging, however, has presented the possibility of directly visualizing individual proteins beyond the diffraction limit of standard fluorescent imaging approaches, including TIRF-M (18), providing a unique platform to probe the functional role of GPCR di/oligomerization at a more detailed molecular level than has been previously possible.

To dissect how GPCR di/oligomerization impacts receptor function and to investigate the role of individual receptor protomers within an oligomer, we have determined the spatial and structural organization of GPCR dimers and oligomers employing LHR^B−^ and LHR^S−^ as tools to create functionally asymmetric complexes. By using dual-color photoactivation localization microscopy with photoactivatable dyes (PD-PALM), we achieve a resolution of ∼8 nm to directly visualize individual GPCR molecules participating in dimers and oligomers at the plasma membrane. PD-PALM demonstrates that LHR is organized in preformed dimeric but primarily diverse oligomeric structures with distinct spatial geometries. Furthermore, altering the functional asymmetry of receptors within an oligomer regulates signal sensitivity and strength. Structural modeling highly aligned with the spatial organizations imaged via PD-PALM and further revealed distinct and complex helix interfaces involved in oligomer formation. The combination of super-resolution imaging with structural modeling provides an unprecedented molecular insight into the complexity and spatial and structural assemblies mediating GPCR oligomerization.

## EXPERIMENTAL PROCEDURES

### 

#### 

##### Materials

Recombinant hCG and LH were purchased from National Peptides and Hormones Program (c/o A. F. Parlow, Harbor-UCLA Medical Center). For PALM studies, CAGE 500 and 552 *N*-hydroxysuccinimide esters for antibody conjugation and direct labeling of receptors were purchased from Abberior. Primary antibodies HA.11 and FLAG were purchased from Covance and Sigma, respectively. For BRET and pRL-cmv luciferase reporter assays, coelentrazine h and coelentrazine, respectively, were purchased from Promega. For cre-luc reporter gene assays, SteadyLite was purchased from PerkinElmer Life Sciences. Fluo-4 direct for calcium imaging was obtained from Invitrogen and HTRF-IP_1_ assay from CisBio.

##### DNA Constructs

Plasmid DNA expressing N-terminally HA-tagged WT LHR, HA-tagged LHR^B−^, and FLAG-tagged LHR^S−^ were generated as described previously (10). For control PD-PALM experiments, N-terminally FLAG-tagged M-CSF receptor was provided courtesy of N. Dibb, Imperial College London, UK. For BRET studies, plasmid DNA encoding *Renilla* luciferase 8 (Rluc8) was kindly provided by S. Gambhir (Stanford School of Medicine), and C-terminally Rluc8-tagged WT LHR, LHR^B−^, and LHR^S−^ were generated by PCR to remove the stop codon and subcloning of the receptors into pcDNA3.1 plasmid containing Rluc8. All constructs were confirmed by sequencing. Plasmid DNA encoding the Gα_s_ and Gα_q_ BRET-tagged construct and the untagged Gβ_1_ and untagged Gγ_2_ were kindly provided by J. Javitch (Columbia School of Medicine, New York) and were generated and validated as described previously (12, 19). The mVenus was utilized for BRET assays, courtesy of A. Miyawaki (RIKEN Brain Science Institute, Japan). cAMP-response element-luciferase (cre-luc) was used for cAMP reporter gene assays, and pRL-CMV transfection control plasmid was purchased from Promega.

##### Cell Culture and Transfections

HEK 293 cells were maintained and cultured as described previously (20). All functional studies were conducted using cell lines stably expressing either HA-WT LHR, HA-LHR^B−^, FLAG-LHR^S−^, or co-expressing HA-LHR^B−^ and FLAG-LHR^S−^. These stable cell lines were generated through Lipofectamine 2000® (Invitrogen)-mediated transfection of the relevant plasmid DNAs, G418 selection, and assessment of cell surface receptor expression by flow cytometry (FACSCalibur, BD Biosciences). All transient transfections were carried out using Lipofectamine 2000® as per the manufacturer's instructions and assayed 48 h post-transfection.

*BRET-*Constitutive and ligand-induced receptor-G protein interactions were assessed by BRET^1^, as described previously (12). Briefly, cells were washed and harvested in PBS and seeded at a density of ∼200,000 cells/well into 96-well plates. To measure constitutive G protein receptor association, coelentrazine h (5 μm) was added, and BRET luminescence and fluorescence ratios were recorded at 475/535 nm for a total of 10 cycles using a FLUOstar (BMG). In parallel cells, fluorescence of the mVenus-tagged Gα_s_ or Gα_q_ protein constructs was determined. For ligand-induced BRET changes, coelentrazine h substrate was added, and the BRET ratio at 475 nm/535 nm was recorded for 1 min. Following this, ligand was added, and the BRET ratio was immediately recorded for a further 1 min. BRET signals were calculated by the dividing the values at 535 nm over that omitted at 475 nm. For constitutive receptor-G protein associations, net BRET values were obtained by subtracting the basal BRET ratio of receptor-tagged Rluc8 alone from all readings. The ligand-induced net BRET changes were calculated by subtracting basal readings from stimulated conditions and further subtracting any changes observed with PBS control. For constitutive receptor-G protein association, duplicate readings were taken, and at least three independent experiments were carried out. For ligand-induced BRET changes, triplicate readings were taken, and 6–11 independent experiments were conducted.

##### Signaling Assays

cre-luc assays were conducted as described previously (10, 20). IP_1_ accumulation was determined using an IP-One HTRF assay kit (CisBio) and measured using a BMG PHERAstar plate reader with HTRF filters. To monitor Ca^2+^ mobilization, Fluo-4 Direct (Invitrogen) labeling was employed as per the manufacturer's instructions, and time-resolved Ca^2+^ mobilization was measured using confocal microscopy. Briefly, cells were loaded with calcium dye for 30 min at 37 °C followed by incubation at room temperature for a further 30 min. Cells were imaged using a TCS-SP5 confocal microscope (Leica) with a ×20 dry objective. Cells were imaged for ∼1 min before agonist treatment, 10 min after agonist addition, and capturing every 1.2 s. Time-lapse movies were analyzed with the Leica LASAF software.

##### PD-PALM

HA.11 and FLAG primary antibodies were labeled with CAGE 552 and CAGE 500 photoswitchable dyes as per manufacturer's instructions (Abberior). Using a derivation of Beer-Lambert Law (*A* = ϵ C.D.), the degree of labeling efficiency was determined for FLAG-CAGE 500 to be 1.0 ± 0.2 dye molecules per antibody and for HA.11-CAGE 552 to be 1.3 ± 0.1 dye molecules per antibody, as per manufacturer's instructions. Cells were plated onto 8-chamber well 1.5 borosilicate coverglass (Labtek) slides. For assessment of basal cell surface receptor molecules, cells were incubated with caged fluorophore-labeled HA.11-CAGE 552/FLAG-CAGE 500 antibodies for direct labeling of receptors in 10% FCS in PBS, at 37 °C, with antibody for 30 min. Cells were washed with PBS and fixed in 4% paraformaldehyde with 0.2% glutaraldehyde for 30 min. The addition of 0.2% glutaraldehyde has been previously shown to dramatically reduce lateral diffusion of transmembrane receptors within the cell membrane, minimizing antibody-induced clustering artifacts that other fixatives can produce (21, 22). Additionally, this fixative method has been previously shown to yield the same minimal clustering artifacts (∼4%) when compared with a nonclustering control (23). Following fixation, cells were washed in PBS and maintained in PBS for imaging. All labeling of receptors was carried out in the dark to ensure minimal photo-switching of labels. Images were acquired using an inverted Axiovert 200 manual inverted wide-field fluorescent microscope (Zeiss, Germany) fitted with a commercial TIRF condenser kit (TILL Photonics GmbH, Germany), with a 1.45 numerical aperture ×100 oil immersion objective. Photo-conversion of CAGE 500 and 552 dyes was achieved with a polychrome light source at 390 nm (Polychrome IV, TILL Photonics GmbH, Uckfield, UK) and was simultaneously imaged and photo-bleached by 491 and 561 nm laser lines, respectively. As the two laser lines have the same optical path through achromatic lenses, chromatic aberrations were negligible. Simultaneous dual channel imaging of CAGE 500 and 552 dyes was achieved using a beam splitter (Optisplit II, Andor) fitted with a T585lp dichroic and ET520-40 and ET632-60 emission filters (all Chroma). The microscope was contained in a plastic draft-proof enclosure maintained at a constant temperature of 25 °C and mounted on a vibration isolation table (Speirs Robertson Corp.). Laser lines were switched on at least 1 h prior to imaging to allow acclimatization and stabilization of the system. These measures served to ensure minimal sample drift throughout the experiments. Each PD-PALM time series was acquired using a cooled electron multiplying charged coupled device camera (EM-CCD; C9100-13, Hamamatsu) and Simple PCI software, with an exposure time of 30 ms. The use of an EM-CCD camera provided a homogeneous image in both imaged channels, also ensuring the integrity of images obtained. Bleed through between the 491- and 561-nm imaged channels was assessed using singly expressing FLAG-LHR^S−^ and HA-LHR^B−^, labeled with CAGE 500 and CAGE 552, and determined to be 3.5 ± 1.0 and 4.3 ± 1.1, respectively. Bright field images of each series were acquired at 108.5-ms exposure and grid images used for post-acquisition alignment of the simultaneously imaged CAGE 500 and 552 channels using Fiji software.

##### Localization Analysis

Localization of receptors detected in 491- and 561-nm channels was individually determined using QuickPALM Fiji plugin (24). Fluorescent intensity images of cropped nonoverlapping areas of 7 × 7 μm within cell borders from corresponding 491- and 561-nm imaged channels were analyzed using the following parameters: a pixel size of 155 nm, a full-width half-maximum value of 3, and signal to noise ratio of 8. Particles were detected if the signal to noise ratio exceeded 8 and full-width half-maximum was 3 pixels or less. Analyzed areas did not span cell membranes to exclude any potential biasing resulting from edge effects. Data tables containing *x-y* particle localization coordinates were generated, and two-dimensional coordinates were determined.

**FIGURE 1.**
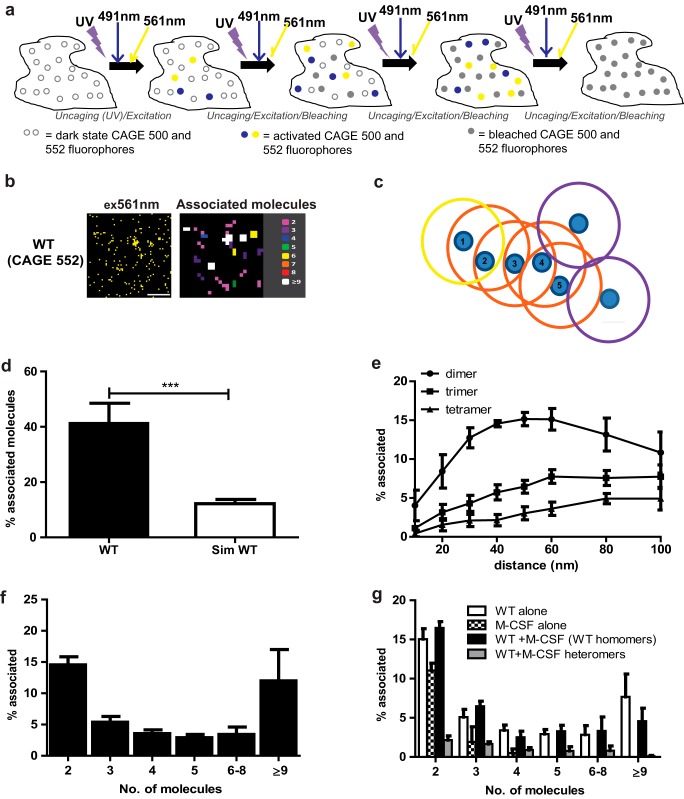
**Visualization of single WT LHR molecules within dimers and oligomers using PD-PALM.** *a,* principles of PD-PALM, utilizing simultaneous dual-color imaging of CAGE 500- and 552-labeled receptors. CAGE 500 and 552 dyes were stochastically “uncaged” by UV, excited, and photo-bleached using 491- and 561-nm lasers, respectively, through multiple cycles until all fluorophores were activated and bleached. *b,* representative reconstructed PD-PALM of the basal landscape of WT LHR stably expressed in HEK 293 cells. Images are 2 μm^2^ from a 7-μm^2^ area. *Scale bars,* 500 nm. Reconstructed data sets were subjected to localization identification using QuickPALM followed by neighborhood analysis, using a 50-nm search radius. A color-coded representative heat map of the number of associated molecules from the respective reconstructed images is depicted. *c,* diagram showing the principle of Getis and Franklin neighborhood analysis. If a receptor (labeled 1, search distance *yellow circle*) was detected within a 50-nm radius of another receptor (labeled 2), then the analysis continued to recursively search for other receptors within a 50-nm radius of the next identified associating receptor (*orange circles*, labeled *3, 4, 5*), until no further associating receptors were detected (*purple circles*). The receptors in this diagram would be identified as a pentamer and two monomers. *d,* percentage of WT LHR dimers, trimers, and tetramers at varying search radii. *e,* comparison of percentage of total associated molecules in cell lines stably expressing WT LHR *versus* randomly generated simulated data sets containing the same density of molecules. *f,* relative proportions of dimeric and higher order oligomeric associated WT LHR. *g,* comparison of WT homomeric, M-CSF receptor homomeric, and WT/M-CSF heteromeric receptor populations following single or co-expression of WT LHR and M-CSF receptor. All data points represent the mean ± S.E. of 10–12 individual cells, *n* = 3. *d* was analyzed by Student's *t* test; *f* by one-way ANOVA with Dunnett's post hoc test; and *g,* by two-way ANOVA with Bonferroni's pairwise comparison post hoc test. ***, *p* < 0.001.

To analyze the number of associated receptor molecules from the *x-y* particle localization coordinates, a custom Java application was designed (PD-Interpreter). The individual files generated using QuickPALM containing localization coordinates of each identified molecule observed in the 491 and 561 nm fields were plotted as an image. A second order Getis Franklin neighborhood analysis was conducted, using a search radius of 50 nm, to determine the degree of both homomeric associations (*i.e.* homo-associating WT LHR, LHR^B−^, or LHR^S−^ protomers) within an individual channel and asymmetric heteromeric associations (*i.e.* LHR^B−^ associating with LHR^S−^) across channels. The analysis worked upon the principle of quantitating the number of molecule(s) within a 50-nm radius of a single identified molecule. To identify dimers and oligomers, the program recursively searched at a 50-nm radius from each associating molecule until no further associating molecules were identified within the allotted search radius (Fig. 1, *a* and *c*). Once an associating group of molecules was assigned, the composition of the di/oligomer was identified and omitted from further searches, so that molecules were not double counted. Data were represented in the form of co-localization plots using differential colors to distinguish 491 and 561 channels and heat maps, with individual colors depicting different numbers of associating molecules. Because of the irreversible photoactivating nature of the CAGE PDs, minimal re-activation or photoblinking of the PDs was observed. However, to discount any potential overestimation of di/oligomers, events within the same channel within a radius of 10 nm of a “parent” activated fluorophore (accounting for a localization precision of 20 nm) were discounted from the analysis. This typically resulted in discounting approximately <1% of activated molecules.

##### Flow Cytometry

Flow cytometry was carried out to quantitate receptor cell surface expression in stable cell lines and transient transfections for BRET analysis, using antibody labeling methods to detect receptor epitope tags as described previously (25). The fluorescence intensity of 10,000 cells was collected for each sample, and treatments were performed in triplicate using a FACSCalibur flow cytometer (BD Biosciences).

##### Spatial Assessment of PD-PALM Images

50 PD-PALM-derived images of trimers and tetramers were randomly selected from 30-s hCG-treated data sets for comparison with computationally derived structural models of LHR^B−^/LHR^S−^ trimers and tetramers. PD-PALM-imaged and structural model-derived spatial arrangements were compared using inter-sphere distances and geometries, and PD-PALM images and structural models containing the same inter-molecule spatial geometries were grouped.

##### Comparative Modeling of the Mouse LHR

The structural model of the mouse LHR was achieved by comparative modeling (using MODELLER software) (26). The structural template consisted of a chimera between the crystal structure of the ECD of the follicle-stimulating hormone receptor (Protein Data Bank code 4AY9) (27) and the structure of constitutively active opsin (Protein Data Bank code 3CAP), with ECD deleted, functioning as an endodomain (28). Specifically, before deleting the opsin-based N terminus (*i.e.* 323–356 sequence), the last four amino acids of the FSHR ECD (*i.e.* the “CEDI” sequence), in particular isoleucine, were fitted onto the corresponding portion of the opsin-based model. Following this, the N terminus was deleted from the endodomain, and the last two amino acids (“DI” sequence) were deleted from the fitted ECD to allow inter-domain junction by comparative modeling. Given the absence of high resolution information on the reciprocal arrangement of the two domains, we think this is a meaningful strategy to adopt. The comparative modeling procedure was carried out as described previously (29). The following disulfide bridges were allowed to form while modeling: Cys-109–Cys-134, Cys-257– Cys-321, Cys-258–Cys-331, Cys-282-Cys-314, and Cys-417–Cys-492. External α-helical restraints were assigned to the 253–273 amino acid stretch belonging to the ill-defined portion of the hinge region. The seventh best model (according to restraint violation) out of 100 was finally selected as also holding one of the best main chain stereochemistry. This model was subjected to refinement of the first extracellular and second intracellular loops (EL1 and IL2, respectively). The best model from such refinement was finally subjected to side chain adjustment when in nonallowed conformations. Because of low resolution, the amino acid segment 283–313 of the hinge region was deleted. The resulting model was used to produce surrogates of the LHR^B−^ and LHR^S−^ forms. The former was indeed a truncation of the LHR^B−^ form (*i.e.* the sequence 1–614) lacking the C-tail, which had no homologous structural template. The LHR^S−^ form consisted of the 1–530 sequence ending at the cytosolic extension of helix 5. Although this may be an oversimplified way to produce the truncated LHR^S−^ form, as it is based on the assumption that it retains the same structure as in the full-length protein, our functional evidence that the LHR^S−^ is able to bind hCG with the same affinity as WT LHR (10) in part supports this assumption.

##### Quaternary Structure Predictions

Prediction of likely architectures of LHR dimers/oligomers followed a computational approach developed for quaternary structure predictions of transmembrane α-helical proteins (30). The approach consisted of rigid-body docking using a version of the ZDOCK program devoid of desolvation as a component of the docking score (31). Because PD-PALM images, in contrast to the crystal structure of the FSHR ECD trimer (Protein Data Bank code 4AY9) (27), suggested the absence or limited contacts between the protomer ECDs, to improve sampling, the N-terminal amino acids 1–334 were not considered in docking simulation. Incidentally, test simulations that did include this sequence, in addition to solutions characterized by inter-ECD contacts, also predicted the same helix contacts inferred from simulations in the absence of the ECD. We first predicted the homo- and heterodimers (*i.e.* LHR^B−^/LHR^S−^ dimer). In the case of homodimerization (*i.e.* LHR^B−/B−^ or LHR^S−/S−^ dimers), two identical copies of the structural receptor model (*i.e.* either in the LHR^B−^ or LHR^S−^ forms) were docked together, with one monomer used as a fixed protein (target) and the other as a mobile protein (probe). For predicting heterodimers, the structural model of LHR^B−^ was taken as a target, whereas the structural model of LHR^S−^ was taken as a probe and vice versa. Default simulation conditions were set, and for each run, the best 4000 solutions were retained and ranked according to the ZDOCK score. The best docked solutions according to the docking score were filtered according to the “membrane topology” filter (using the FIPD software) (30), discarding all solutions that violated the membrane topology requirements. The membrane topology filter discards all the solutions characterized by a deviation angle from the original *z* axis, *i.e.* tilt angle, and a displacement of the geometrical center along the *z* axis, *i.e. z*-offset, above defined threshold values. In the case of LHR, the tilt angle and the *z*-offset thresholds were defined as 0.4 radians and 6.0 Å, respectively. The filtered solutions from each run were merged with the target protein, leading to an equivalent number of dimers that were clustered using a Cα-root mean square deviation threshold of 3.0 Å for each pair of superimposed dimers. All the amino acid residues in the dimer were included in Cα-root mean square deviation calculations. Cluster analysis was based on a Quality threshold-like clustering algorithm (32), implemented both in the FIPD and Wordom software (30, 33). Because the filtering cutoffs of the membrane topology parameters were intentionally quite permissive, inspection of the cluster centers (*i.e.* the solutions with the highest number of neighbors in each cluster) served as a final filter to discard remaining false positives, leading to a reduction of the reliable solutions to about 1% of the total 4000 solutions. The best scored docking solutions from the most populated and reliable cluster(s) was(were) finally chosen. Predictions of higher order oligomers (*i.e.* trimers and tetramers) were carried out essentially by running a new docking simulation using the predicted dimer/trimer as a target and the protomer as a probe. As for tetramerization, dimer *versus* dimer docking was also carried out. Filtering and cluster analysis then followed the same procedure as the one employed for predicting dimers. In summary, 4 runs of dimerization, 31 runs of trimerization, and 174 runs of tetramerization led to selection of 8 homodimers, 8 heterodimers, 12 homotrimers, 27 heterotrimers, and 86 heterotetramers holding a reliable membrane topology and absence/minimal steric clashes between the N termini. A sub-selection of a few representatives holding nonredundant architectures was determined.

##### Statistical Analysis

All basal and ligand-dependent PD-PALM data represent the mean ± S.E. for 10–12 cells from three independent experiments. To assess differences in basal receptor associations, a two-way ANOVA with Bonferroni's multiple comparison post hoc test was conducted, comparing the number of associated molecules, within the receptor cell line. For ligand-induced effects, a two-way ANOVA with Bonferroni's multiple comparison post hoc test was also conducted, analyzing the impact of ligand treatment with the associated receptor forms. cre-luc and IP_1_ concentration-dependent curves were subjected to two-way ANOVA and Dunnett's post hoc test. Maximum cre-luc, IP_1_, and Ca^2+^ response plots were analyzed by paired Student's *t* test. For cre-luc, IP_1_, and Ca^2+^ studies, each data point represented the mean ± S.E. of at least three independent experiments, conducted in triplicate. Correlation analysis of receptor ratio and functional activity was carried out using Pearson's correlation. Receptor ratio *versus* the composition of oligomeric complexes was analyzed using a one-way ANOVA with Dunnett's post hoc test.

All statistical evaluations were performed using GraphPad Prism Version 5 (San Diego, CA), and significance was determined as a probability value of *p* < 0.05.

## RESULTS

### 

#### 

##### Visualization of LHR Oligomers Utilizing Photoactivated Fluorophores for Dual-color PD-PALM

To determine the molecular complexes that LHR forms under conditions of designed functional asymmetry, we employed PALM with TIRF-M to visualize single receptor molecules at the plasma membrane. PALM commonly utilizes photoactivatable proteins (*e.g.* mEOS and Dendra). However, to specifically analyze cell surface receptor complexes, we utilized our previously characterized extracellular N-terminally epitope-tagged LHRs, FLAG and HA, and CAGE photoactivatable dyes (PD) to directly label HA and FLAG primary antibodies, an approach we termed PD-PALM. PDs are advantageous as they are brighter and more photo-stable than most photoactivatable proteins, and therefore they enhance the accuracy of identifying and localizing activated fluorophores. Importantly, PDs undergo irreversible activation and bleaching (34), and so they were an ideal choice for our quantitative studies to decipher the molecular dimeric and oligomeric composition of LHR. With PD-PALM, CAGE dyes are “masked” in the dark state (Fig. 1*a*). Illumination with UV light rapidly “unmasks” PDs allowing detection followed by photo-bleaching. PDs undergo stochastic excitation to ensure only a small subset of molecules are activated at any given time. This cycle is repeated multiple times to release and capture all unactivated molecules, and the resulting image is compiled through localization analysis, resolving individually activated molecules from defined parameters, and compressed into a single plot from the sequentially imaged frames.

Before determining the molecular complexes formed between intermolecular cooperating LHR mutants, we first analyzed the basal cell surface organization of wild type (WT) LHR. HEK 293 cells stably expressing HA-tagged WT LHR were generated and utilized. The expression level of WT LHR, as quantitated by PD-PALM, was within the cell surface expression range (2000–8000 receptors/cell) of that previously reported for ovarian and testicular LHR (4000–2,000 receptors/cell) (35, 36) and so expressed within a physiological range for this receptor. HA-tagged WT LHR was labeled with directly conjugated CAGE 552-HA antibody (Fig. 1*b*). The degree of labeling was determined to be 1.3 ± 0.1 PD/antibody, suggesting ∼1:1 labeling of PD to antibody (see “Experimental Procedures” for further details). Saturation of antibody binding was confirmed by comparing labeling post-fixation at 4 °C overnight *versus* labeling live cells at 37 °C for 30 min followed by fixation. The total percentage of detected associated molecules labeled overnight in fixed samples yielded ∼37% for 50–80 receptors/μm^2^ *versus* ∼41% for 60–85 receptors/μm^2^ for samples that were labeled in live cells for 30 min. This indicated that saturation of antibody labeling was reached using our chosen antibody labeling approach. Moreover, comparison with pre- and post-fixation labeling also demonstrated that the complexes observed were not due to antibody-induced clustering of molecules. The fixation methodology employed was selected based on prior characterization to yield minimal clustering artifacts (see under “Experimental Procedures” for further details). To quantitate the number of associating molecules, we used a modified Getis and Franklin second-order neighborhood analytical approach (Fig. 1*c*) (37). This approach has been previously validated for assessing associating molecules in single and two channel PALM data sets (38). For the analysis, a search radius of 50 nm was selected, based on the sum of the following criteria: the localization precision calculated to be ∼20 nm using Thompson's criteria (39); the size of the large N-terminal extracellular domain (ECD) of LHR; and the maximum distance that antibody labeling has been reported to provide, of ∼15–20 nm per antibody (40). We additionally tested the robustness of using this methodology for analyzing the propensity of LHR to dimerize and oligomerize using simulated data sets (Fig. 1*d*). The use of the simulated random data sets served to test whether experimentally observed trends of receptor protomer associations were attributable to an increased propensity of WT LHR with which to associate or whether they reflected the expected number of associated molecules of randomly distributed rather than ordered data. Comparison of the total number of associated molecules observed from the experimentally determined WT LHR associations and the randomly generated simulated data associations revealed a significantly higher number of associated WT LHR (Fig. 1*d*). This demonstrated that the criteria used for the analyses were sufficiently stringent to distinguish the difference between expected mathematically predicted associations of random data sets and more highly ordered receptor associations observed with the WT LHR. The selected search radius also identified the highest number of lower order associating dimers/trimers/tetramers (Fig. 1*e*). Analysis of CAGE 552-HA-labeled WT LHR in the basal state revealed a diverse makeup of monomers, dimers, and oligomers (Fig. 1, *b* and *f*). Quantitative analysis of WT LHR complexes revealed that 41.4 ± 7.1% were associated as di/oligomers (Fig. 1, *d* and *f*). The number of dimers observed were 14.6 ± 1.1%, with the majority of associating receptors, 26.8 ± 6.7%, forming oligomers (Fig. 1*f*). As a control for the specificity of receptor associations observed with LHR, and for our labeling and analytical methodology, we measured the ability to detect self-association of a tyrosine kinase receptor for colony-stimulating factor-1 (M-CSF) that is predominantly monomeric in the basal state, unlike many GPCRs. M-CSF receptor was N-terminally FLAG-tagged and labeled with CAGE 500-FLAG. The number of cell surface M-CSF receptors observed (∼60–80 receptors/μm^2^) was similar to the number of WT LHR imaged by PD-PALM. Quantitation of M-CSF receptor associations revealed that 85.6 ± 1.9% were monomeric, with only 11.0 ± 0.9% existing as pre-formed homodimers (Fig. 1*g*). In contrast to the WT LHR, no complexes with >4 associated molecules were observed, consistent with the concept that in the basal state tyrosine kinase receptors are primarily monomeric (41). Furthermore, this also confirmed that our antibody labeling and fixation methods resulted in minimal clustering artifacts, adding further confidence to the validity of our chosen labeling methods and data observed. Co-expression of M-CSF with WT LHR did not affect the number or nature of homo-associated complexes of LHR. Moreover, minimal (<2%) hetero-complexes were detected between WT LHR and M-CSF receptors (Fig. 1*g*), further demonstrating the specificity of receptor-receptor associations observed by antibody-based PD-PALM and of our chosen analytical parameters.

**FIGURE 2.**
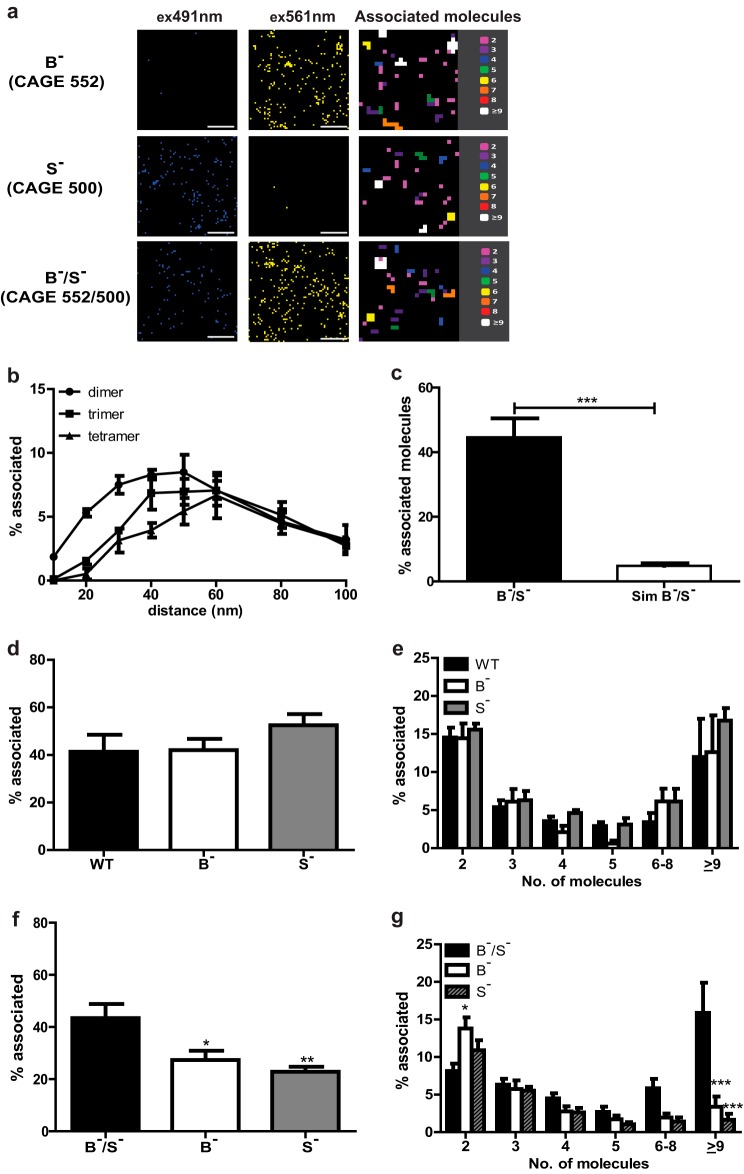
**Visualization of LHR^B−^/LHR^S−^ dimers and oligomers using PD-PALM.** *a,* representative reconstructed PD-PALM images in 491- and 561-nm channels of singly expressed LHR^B−^ (B^−^), LHR^S−^ (S^−^), and co-expressing LHR^B−^/LHR^S−^ (B^−^/S^−^) stable cell lines. Images are 2 μm^2^ from a 7-μm^2^ area. *Scale bars,* 500 nm. Reconstructed data sets were subjected to localization identification using QuickPALM followed by neighborhood analysis, using a 50-nm search radius. A color-coded representative heat map of the number of associated molecules from the respective reconstructed images is depicted. *b,* percentage of LHR^B−^/LHR^S−^ dimers, trimers, and tetramers at varying search radius used for the nearest neighborhood analysis. Each data point represents the mean ± S.E. of three independent experiments. *c,* comparison of the total number of associated molecules observed in cell lines co-expressing LHR^B−^/LHR^S−^ and randomly generated simulated data sets containing the same range of molecule densities. *d,* comparison of the total number of WT-, LHR^B−^-, and LHR^S−^-associating molecules in cell lines singly expressing these receptor subtypes. *e,* relative proportions of dimeric and higher order oligomeric associated WT, LHR^B−^, and LHR^S−^ from the total number of associated receptors in *d. f,* comparison of the percentage of total associated heteromeric LHR^B−^/LHR^S−^, homomeric LHR^B−^, and LHR^S−^ in cell lines stably expressing LHR^B−^/LHR^S−^. *g,* relative proportions of LHR^B−^/LHR^S−^ heteromers, and LHR^B−^ and LHR^S−^ homomers within the LHR^B−^/LHR^S−^-expressing cell line from *f*. *, *p* < 0.05; **, *p* < 0.01; ***, *p* < 0.001.

For PD-PALM imaging of cells expressing HA-LHR^B−^ and/or FLAG-LHR^S−^, CAGE 552-HA and 500-FLAG were employed, respectively. The labeling efficiency of CAGE 500-FLAG was 1.0 ± 0.2 PD/antibody. Minimal channel bleed through was detected with receptors labeled with either CAGE 552- or CAGE 500-conjugated antibodies (Fig. 2*a*, CAGE 500 = 3.5 ± 1.0% and CAGE 552 = 4.3 ± 1.1%). The robustness of our chosen analytic parameters was also tested for two-color PD-PALM. As with WT LHR, the search radius of 50 nm also identified the highest number of lower order associations of LHR^S−^/LHR^B−^ in cell lines stably co-expressing these receptors (Fig. 2*b*). Additionally, comparison of the percentage of associated molecules observed in cell lines co-expressing LHR^S−^/LHR^B−^ with randomly generated two-channel simulated data sets showed a significantly higher proportion of LHR^S−^/LHR^B−^-associated molecules via PD-PALM than would be randomly expected (Fig. 2*c*). Stable cell lines singly expressing HA-LHR^B−^ or FLAG-LHR^S−^, at equivalent surface levels to cells stably expressing WT LHR, did not significantly differ in the percentage of total associated receptors (Fig. 2*d*) or the composition of homomeric complexes (Fig. 2*e*) compared with WT LHR indicating that the receptor mutations did not affect their ability to self-associate. In cells stably co-expressing HA-LHR^B−^ with FLAG-LHR^S−^ (LHR^B−^/LHR^S−^) (Fig. 2*a*, *lower panel*), the percentage of total associated molecules was similar to that observed with WT and singly expressed mutant receptors (Fig. 2, *d* and *f*). Interestingly, the number of homomers of either LHR^B−^ or LHR^S−^ was significantly reduced in cells co-expressing LHR^B−^/LHR^S−^ (Fig. 2*f*). Within the same cells, there was a significant decrease in the number of LHR^B−^/LHR^S−^ dimers compared with LHR^B−^ dimers and an increase in the number of LHR^B−^/LHR^S−^ oligomers (Fig. 2*g*). Overall, this suggests that LHR mutants preferentially associate as LHR^B−^/LHR^S−^ oligomeric complexes.

##### Intermolecular Cooperation Is Sufficient to Fully Activate hCG but Not LH-mediated G protein Signaling

To determine whether the preferential association of LHR^B−^-LHR^S−^ oligomeric complexes impacted on receptor signaling, we assessed whether LHR intermolecular cooperation was sufficient to mediate signaling via distinct G protein-dependent pathways activated by LHR. LHR has two endogenous ligands, luteinizing hormone (LH) and its highly active analog human chorionic gonadotropin (hCG). Previous studies have demonstrated that intermolecular cooperation via LHR^B−^/LHR^S−^ can activate hCG-mediated cAMP signaling *in vitro* (8, 9, 42, 43) and endogenous LH responses in male mice *in vivo* (10). Like many GPCRs, LHR can couple to additional G proteins, namely Gα_q/11_, under conditions of high receptor and/or ligand concentrations, as demonstrated in the ovarian follicle and are thought to be essential to induce ovulation (44).

**FIGURE 3.**
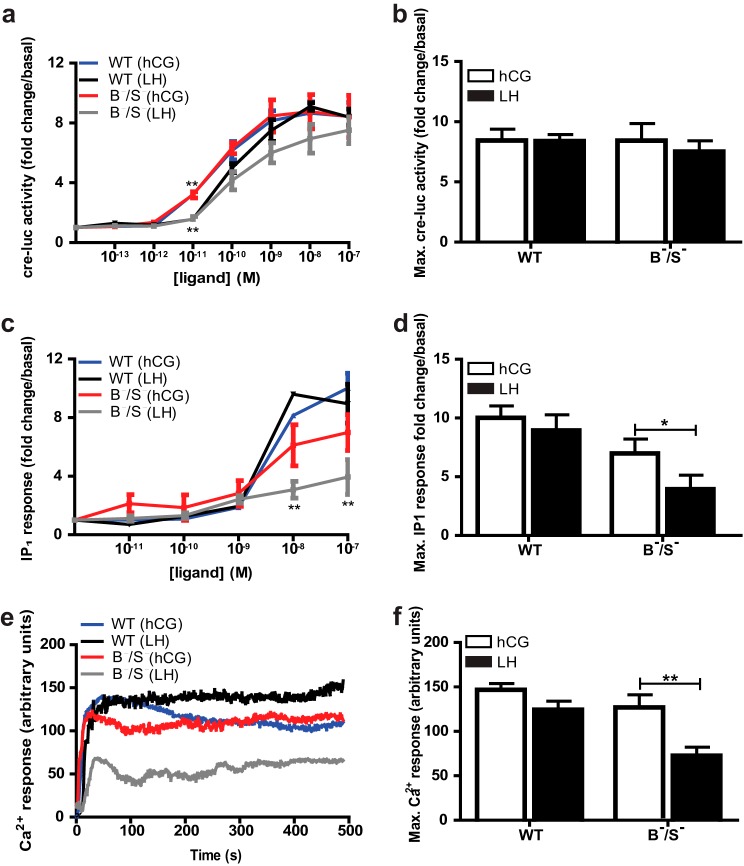
**Intermolecular cooperation of LHR is sufficient to fully activate hCG but not LH-mediated G protein signaling.** *a,* HEK 293 cells stably expressing WT LHR (*WT*) or LHR^B−^/LHR^S−^ (B^−^/S^−^) transiently transfected with cre-luc reporter gene and *Renilla* luciferase (normalization control) and treated with hCG or LH (10^−13^–10^−7^ m) for 4 h. *b,* maximum cre-luc responses to hCG and LH in WT and LHR^B−^/LHR^S−^ cell lines from *a. c,* total IP_1_ measured in cells stably expressing WT LHR or LHR^B−^/LHR^S−^ following stimulation with either hCG or LH (10^−12^–10^−7^ m) for 60 min. *d,* maximum IP_1_ responses to hCG or LH in WT and LHR^B−^/LHR^S−^ (*c*). Concentration responses (*a* and *c*) were normalized to individual basal responses and expressed as fold change/basal, each data point represents mean ± S.E., *n* = 3. *e,* representative Ca^2+^ fluorescent intensity plots (arbitrary units) from real time confocal imaging with Ca^2+^ dye, Fluo-4 Direct, following stimulation with LH or hCG (100 nm). Responses were obtained from 10 individual cells, *n* = 5. *f,* maximum Ca^2+^ responses from *e* following treatment with LH or hCG.

Cells expressing WT LHR or LHR^B−^/LHR^S−^ were stimulated with either LH or hCG, and cAMP signaling was measured by a cAMP-response element (cre) luciferase reporter gene. Responses were equipotent, with comparable concentration curves and maximal responses when comparing WT LHR and LHR^B−^/LHR^S−^ (Fig. 3, *a* and *b*). Similar maximal responses were also obtained using a Glo-Sensor cAMP assay (data not shown). Differences were observed in the potency of the two ligands in both cell lines (Fig. 3*a*), consistent with known *K_d_* values (45). Ligand-dependent LHR activation of Gα_q/11_-PLC signaling was determined by measurement of IP_1_ and Ca^2+^. Treatment of cells expressing WT LHR or LHR^B−/^LHR^S−^ with hCG induced similar maximal activation of Ca^2+^ and IP_1_ (Fig. 3, *c–f*). The higher concentrations of ligand required to induce IP_1_ responses (Fig. 3*c*) are consistent with the known high receptor and high ligand concentrations necessary for coupling of LHR to Gα_q/11_ (46). However, in cells expressing LHR^B−^/LHR^S−^, LH-dependent increases in IP_1_ and Ca^2+^ were significantly attenuated compared with hCG, indicating that intermolecular cooperation was insufficient for full LH-dependent Gα_q/11_-PLC signaling (Fig. 3, *c–f*).

**FIGURE 4.**
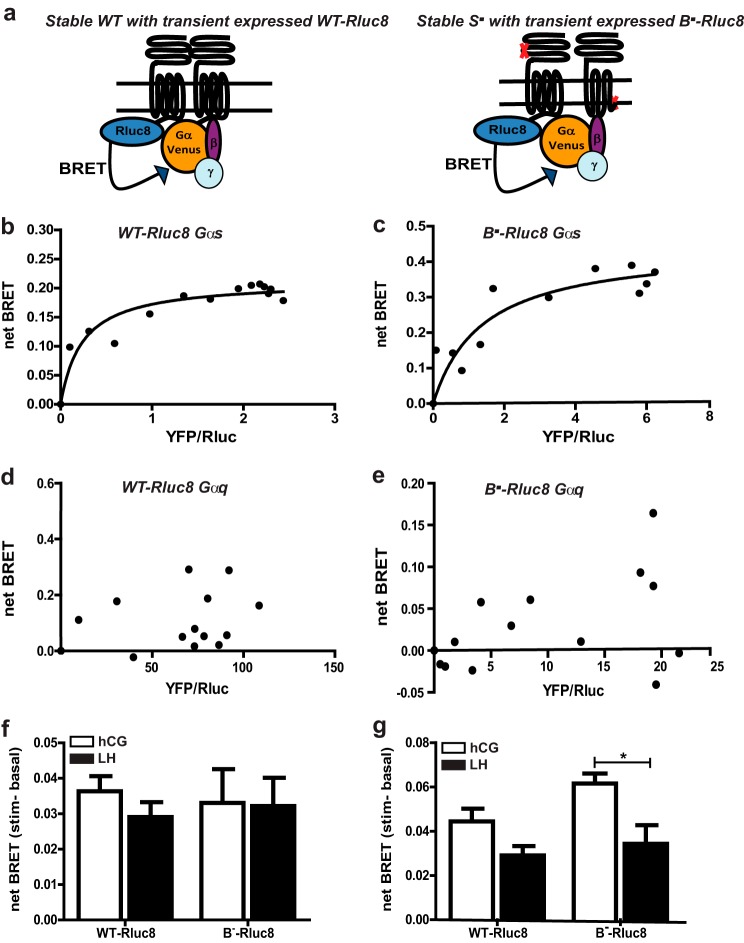
**Constitutive and ligand-induced BRET between WT LHR or LHRB^−^ with either Gα_q_ or Gα_s_ proteins.** *a,* schematic showing the experimental design of receptor-G protein associations. Gα proteins were Venus-tagged, and β and γ subunits were untagged. Representative BRET saturation curves were used to measure constitutive association of WT-Rluc8 with Gα_s_ (*b*), LHR^B−^Rluc8 with Gα_s_ (*c*), WT-Rluc8 with Gα_q_ (*d*), or LHR^B−^Rluc8 with Gα^q^ (*e*). Data are representative of at least three independent experiments, conducted in duplicate. Cells stably expressing WT LHR or LHR^S−^ were transiently transfected with Venus-tagged Gα_s_ (*f*) or Gα_q_ (*g*) and either LHR WT-Rluc8 (in cell lines stably expressing WT) or LHR^B−^Rluc8 (in cell lines stably expressing LHR^S−^). Basal BRET was obtained for 1 min prior to LH or hCG (100 nm) addition for 1 min. *f* and *g* represents mean ± S.E. of 6–11 independent experiments conducted in triplicate. *, *p* < 0.05.

To determine whether this difference in Gα_q/11_-PLC signaling was due to a direct effect on receptor/G protein-coupling, BRET was used to measure ligand-induced associations of Rluc8-tagged WT LHR or LHR^B−^ with Venus-tagged Gα_s_ or Gα_q_ in intact live cells (12, 19). WT LHR-Rluc8 was transiently expressed in the WT LHR stable cell line to directly compare with experimental conditions employed to measure BRET signals for LHR^B−^-Rluc8 expressed in the LHR^S−^ stable cell line (Fig. 4*a*). The LHR^B−^ was used as the donor-tagged receptor as this protomer provided the interface for G protein signaling, whereas the LHR^S−^ protomer binds ligand only. Constitutive association of WT LHR-Rluc8 and LHR^B−^Rluc8 with Venus-Gα_s_ was observed, but not with Venus-Gα_q_ (Fig. 4, *b--e*). Treatment of WT LHR-Rluc8-expressing cells with LH or hCG induced comparable net BRET increases between the receptor and either G protein over mock-treated PBS controls (Fig. 4, *f* and *g*), consistent with the signaling responses observed. Stimulation with LH or hCG produced similar net BRET signals between LHR^B−^ Rluc8 and Gα_s_-Venus (Fig. 4*f*). In contrast, treatment with LH resulted in a significantly lower net BRET signal between LHR^B−^ Rluc8 with Gα_q_-Venus compared with hCG-dependent responses (Fig. 4*g*).

**FIGURE 5.**
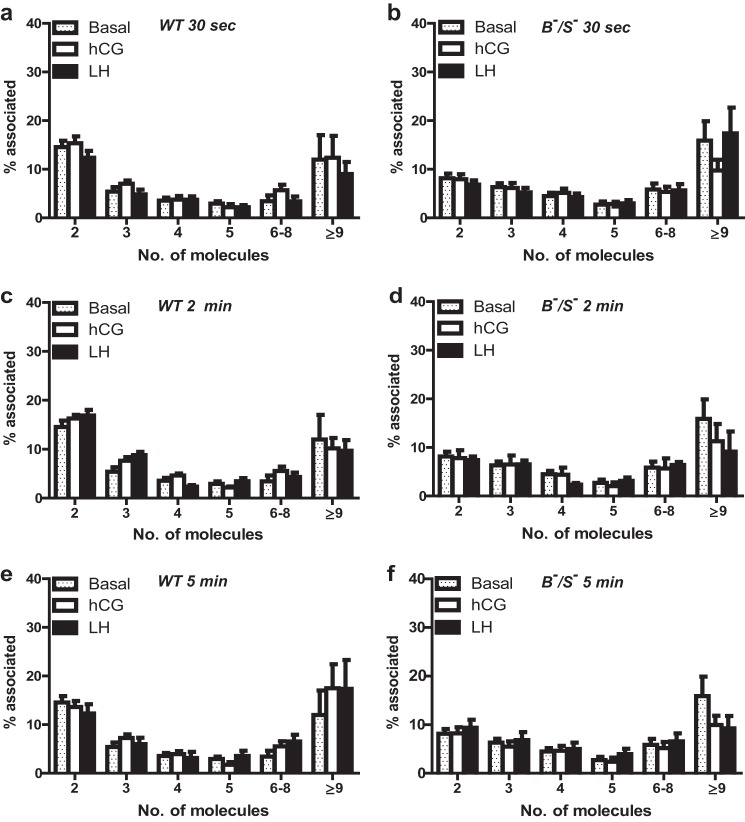
**Organization of WT LHR and LHR^B−^/LHR^S−^ complexes following ligand treatment.** Cells stably expressing either WT LHR or LHR^B−^/LHR^S−^ were treated with hCG and LH (both 10 nm) following 30 s (*a* and *b*), 2 min (*c* and *d*), or 5 min (*e* and *f*). Samples were imaged via PD-PALM, and relative proportions of dimeric and higher order oligomeric associated receptors were quantitated as in Fig. 1. Each data point represents the mean ± S.E. of 8–12 individual cells from three independent experiments. A two-way ANOVA with Bonferroni's post hoc test was conducted.

The differential ability of LHR intermolecular cooperation to couple and activate distinct G protein pathways in response to LH, but not hCG, may suggest potential ligand-dependent differences in the LHR^B−^/LHR^S−^ complexes formed. As localization microscopy requires multiple cycles of fluorophore activation over time, live temporal analysis was not feasible and would compromise image resolution. Therefore, cells were treated at different time points with either ligand and rapidly fixed to capture any potential ligand-dependent changes in receptor organization. In cells expressing WT LHR or LHR^B−^/LHR^S−^, treatment with LH or hCG for 30 s (Fig. 5, *a* and *b*), 2 min (Fig. 5, *c* and *d*), or 5 min (Fig. 5, *e–f*) did not significantly change the proportion of dimeric or oligomeric complexes observed when compared with basal within a specific time point. Additionally, comparison of activation with both ligands had no significant effect on the proportions of preformed complexes for either WT LHR or LHR^B−^/LHR^S−^ populations (Fig. 5, *a–f*) as measured under these conditions.

##### Ratiometric Composition of LHR^B−^ to LHR^S−^ Complexes Impacts Signal Sensitivity

**FIGURE 6.**
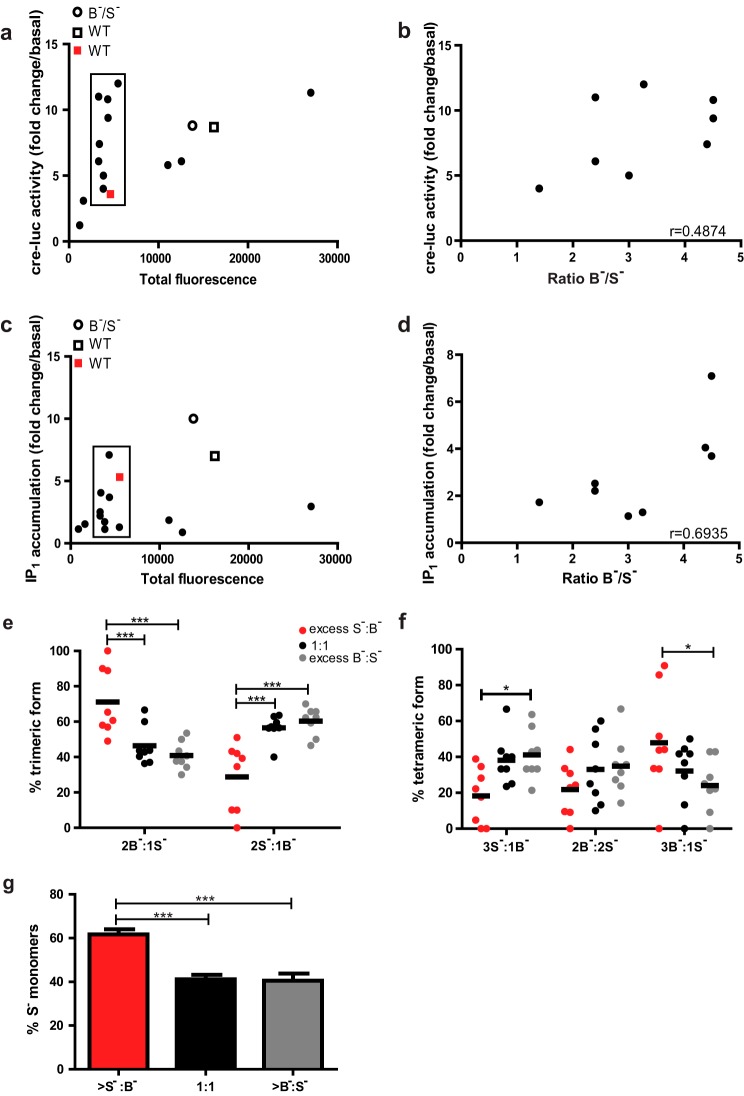
**Ratiometric molecular composition of LHR^B−^ to LHR^S−^ impacts signal sensitivity and oligomeric composition.** Comparison of hCG-dependent cre-luc activity (*a*) and IP_1_ accumulation (*b*) in cells stably expressing LHR^B−^/LHR^S−^ at differing cell surface densities, as determined by flow cytometry and depicted as mean fluorescence. *Open circle* and *square* depict relative values for WT LHR (*WT*) and LHR^B−^/LHR^S−^ (B^−^/S^−^) cell lines, respectively, used for all ligand-induced signaling and PD-PALM experiments. The *red square* depicts a cell line stably expressing WT at equivalent expression levels and relative cre-luc and IP_1_ functional activities. A Pearson's correlation of the cell lines expressing LHR^B−^/LHR^S−^ at similar overall surface levels (*highlighted black box, filled circles*) was carried out comparing cre-luc activity (*c*) and IP_1_ accumulation (*d*) to the cell surface ratio of LHR^B−^/LHR^S−^ as determined by flow cytometry analysis. Each data point represents the mean ± S.E. of three independent experiments, completed in triplicate. *e–g,* analysis of basal PALM datasets to determine how the ratio of LHR^S−^/LHR^B−^ within an individual cell impacts on the molecular ratiometric composition of trimeric (*e*) and tetrameric (*f*) complexes. *g,* effect of the ratiometric composition of LHR^B−^/LHR^S−^ on the percentage of LHR^S−^ monomers in cells with excess LHR^S−^/LHR^B−^, a 1:1 ratio or an excess LHR^B−^/LHR^S−^. Each data point in *e–g* represents a 7-μm^2^ area from an individual cell, for a total of eight cells from four independent experiments. Data in *e* and *f* were subjected to a two-way ANOVA with Bonferroni's multiple comparison post hoc test; *g,* one-way ANOVA with Bonferroni's pairwise comparison post hoc test. *, *p* < 0.05; ***, *p* < 0.001.

Although the total level of surface receptors expressed in the LHR^B−^/LHR^S−^ stable cell line was equal to WT LHR cells, there was a greater proportion of surface LHR^S−^ to LHR^B−^ by ∼3-fold (WT 320 arbitrary units *versus* LHR^S−^/LHR^B−^ 254/81 arbitrary units). We therefore asked whether altering the ratio of surface LHR^S−^ to LHR^B−^ impacted the signaling response and composition of oligomeric complexes observed by PD-PALM. A further 13 stable cell lines co-expressing each mutant receptor at varying ratios were created and tested for hCG-dependent cAMP/cre-luc activation and IP_1_ accumulation. A wide range of hCG-induced responses were observed (Fig. 6, *a* and *c*), and comparison of signaling responses in cell lines with similar total surface receptor fluorescence showed highly varied signal profiles (Fig. 6, *a* and *c, boxed symbols*). When the ratio of LHR^S−^ to LHR^B−^ in these cell lines was correlated with cre-luc or IP_1_ responses, there was a positive correlation between an excess of LHR^S−^ to LHR^B−^ with an increased signal response (Fig. 6, *b* and *d*), indicating that signal sensitivity could be regulated by the ratio of LHR^S−^ to LHR^B−^ within a complex.

The increased signaling observed under the conditions of increasing surface expression of LHR^S−^ suggests the asymmetric composition of mutant receptors within an individual LHR^B−^/LHR^S−^ oligomer have higher ratios of LHR^S−^ to LHR^B−^. To directly determine this, we visualized the composition of trimeric and tetrameric LHR^B−^/LHR^S−^ oligomers by PD-PALM under conditions of specific ratiometric surface levels of LHR^S−^ to LHR^B−^. We focused on lower order oligomers for this analysis as the formation of trimers and tetramers displayed a weak or no correlation with receptor density (*r* = 0.40 for trimers; *r* = 0.26 for tetramers). Similarly, lower order oligomers of WT LHR (<5) displayed weak correlation with receptor density (*r* = 0.325). Unexpectedly, in cells expressing an excess of cell surface LHR^S−^, there was a significantly higher proportion of LHR^B−^/LHR^S−^ trimers and tetramers that included a greater number of LHR^B−^ protomers (>S:B, *red dots*, Fig. 6, *e* and *f*). Moreover, a strong positive correlation was observed between increasing expression levels of LHR^S−^ and the number of 2B^−^:1S^−^ trimers (*r* = 0.98). There was also a significant increase in the percentage of LHR^S−^ monomers in cells expressing an excess of LHR^S−^ (Fig. 6*g*), indicating that LHR^S−^ preferentially associates with LHR^B−^ over self-association. As we observed an increase in signaling under such conditions, yet we have previously shown that either LHR^B−^ or LHR^S−^ alone is inactive (10), this suggests that formation of LHR complexes containing an excess of heterotrimeric G protein-binding protomers (LHR^B−^) may increase the sensitivity of ligand-induced cellular signaling responses.

##### Spatial and Structural Signatures of Lower Order LHR^B−^/LHR^S^ Complexes

**FIGURE 7.**
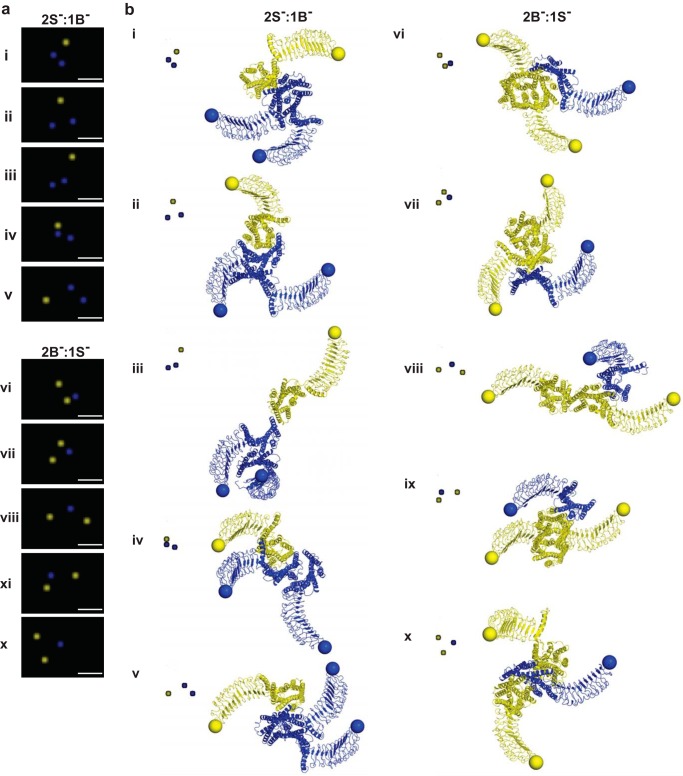
**PD-PALM imaging and structural modeling of functionally asymmetric trimeric complexes.** *a,* representative PD-PALM images showing spatial arrangements of LHR^B−^ (*yellow*) and LHR^S−^ (*blue*); *scale bars* are 50 nm. 50 PD-PALM identified trimers were used for comparison of spatial arrangements with computationally derived structural data (see “Experimental Procedures” for further information). *b,* structural models of predicted heterotrimers (B^−^/S^−^). Structural modeling of heterotrimer architectures that closely aligned with PD-PALM images in *a*. The structures are depicted from the extracellular side in a direction perpendicular to the membrane surface. *Yellow* and *blue spheres* are centered on the Cα-atom of the first N-terminal amino acid, and *yellow* and *blue* colors indicate LHR^B−^ and LHR^S−^, respectively. *Roman numerals* in *a* and *b* indicate matched complexes. The *inset* images are color-inverted images (from *black* to *white*) of respective PD-PALM image as listed in *a* and enlarged to double the size of the original PD-PALM images shown in *a*.

**FIGURE 8.**
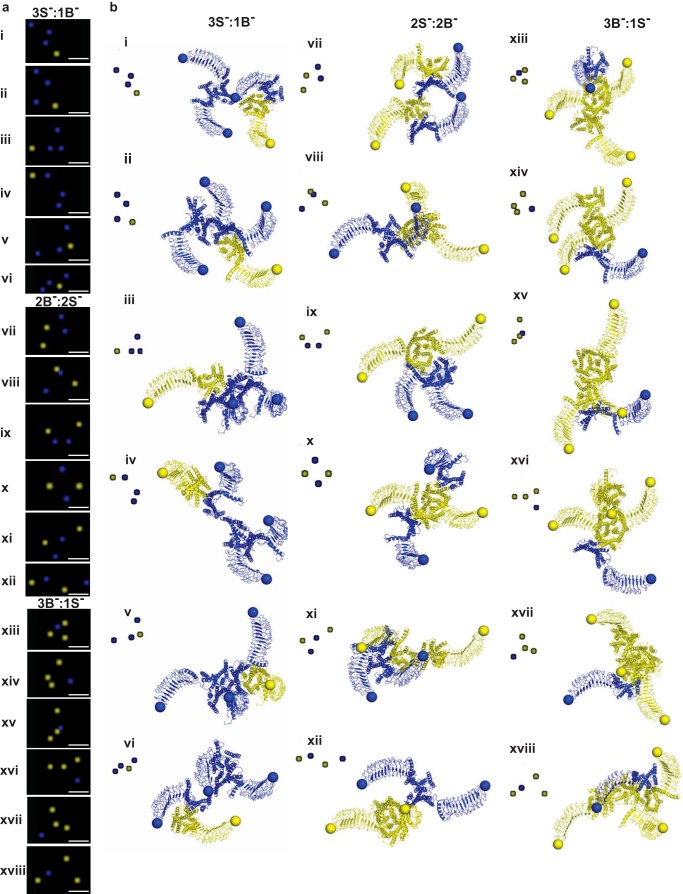
**PD-PALM imaging and structural modeling of functionally asymmetric tetrameric complexes.** *a,* representative PD-PALM images showing spatial arrangements of LHR^B−^ and LHR^S−^. *Scale bars,* 50 nm. 50 PD-PALM imaged tetramers were selected and used for comparison of spatial arrangements with structural data (see “Experimental Procedures” for further details). *b,* structural models of the predicted heterotetramers (B^−^/S^−^). Representations of the heterotetramer architectures that closely aligned with PD-PALM resolved images in *a*, imaged from the extracellular side perpendicular to the membrane surface. *Yellow* and *blue* colors indicate LHR^B−^ and LHR^S−^ forms, respectively. *Roman numerals* in *a* and *b* indicate matched complexes. *Spheres* are centered on the Cα-atom of the first amino acid. The *insets* are derived from color inverting and enlarging by 2-fold with respect to PD-PALM images from *a*.

To understand how LHR^B−^/LHR^S−^ oligomers are formed at a detailed molecular level in intact cells, we resolved the spatial arrangement of individual LHR^B−^/LHR^S−^ trimers and tetramers as visualized by PD-PALM, combined with molecular modeling. The localization of molecules identified by PD-PALM represents the dye position of the antibody-bound N terminus, providing information on the arrangements of trimers and tetramers but not structural information on inter-protomer interfaces in these complexes. Thus, docking simulations on surrogates of LHR^B−^ and LHR^S−^ were used to predict both spatial organization and interacting interfaces of protomers within an oligomer. The structural models were achieved by comparative modeling using the two available crystal structures of the related glycoprotein hormone receptor, the FSHR ECD (27, 47) and crystal structures of constitutively active opsin as templates (see under “Experimental Procedures” for further details on models). The employment of opsin served to approximate the active state of LHR (our preliminary modeling studies comparing rhodopsin (basal state) and opsin showed substantial similarities in predicted interacting interfaces (data not shown)). Trimers visualized by PD-PALM were primarily organized in triangular formations (Fig. 7*a*), although tetramers exhibited a higher variety of distributions but were still comprised of at least one triangular sub-arrangement (Fig. 8*a*). Unexpectedly, the predicted spatial organization by structural modeling, based on the first amino acid of the N terminus (*yellow* or *blue sphere* centered on the Cα-atom) closely matched PD-PALM resolved geometries of trimers and tetramers (Figs. 7*b* and 8*b*), with >90% of PD-PALM imaged complexes aligning with structural predictions. Differences were observed in absolute inter-molecule values identified by PD-PALM and structural models, with PD-PALM distance ranging from 4 to 90 nm and structural models from 4 to 20 nm. This difference in distances is most likely due to the PD-conjugated primary antibody bound to the N-terminal epitope tag employed in the PD-PALM imaged data compared with the Cα sphere of the N terminus of LHR^B−^ or LHR^S−^ resolved by structural analysis.

Spatial organization of the intermolecular cooperating complexes were dictated by contacts between helices and the distinct shape of the large N terminus. Changes in the conformation of the hinge region, which determines the orientation of the ECD with respect to the endodomain, may also contribute to the geometrical shaping of the di/oligomers. However, the strong integration of the hinge region into the rigid hormone-binding leucine-rich repeat region (27) makes this possibility quite remote. The quaternary structure of the helix bundles of each protomer indicates that the arrangements of receptors in trimers and tetramers are primarily linear (Fig. 8*b, panels iv, vi,* and *viii*). Therefore, spatial organizations of the receptors inferred from PD-PALM and structural modeling are due to the rigid and defined shape of the ECD, in line with the crystallographic complex of the FSHR ECD trimer (27).

**FIGURE 9.**
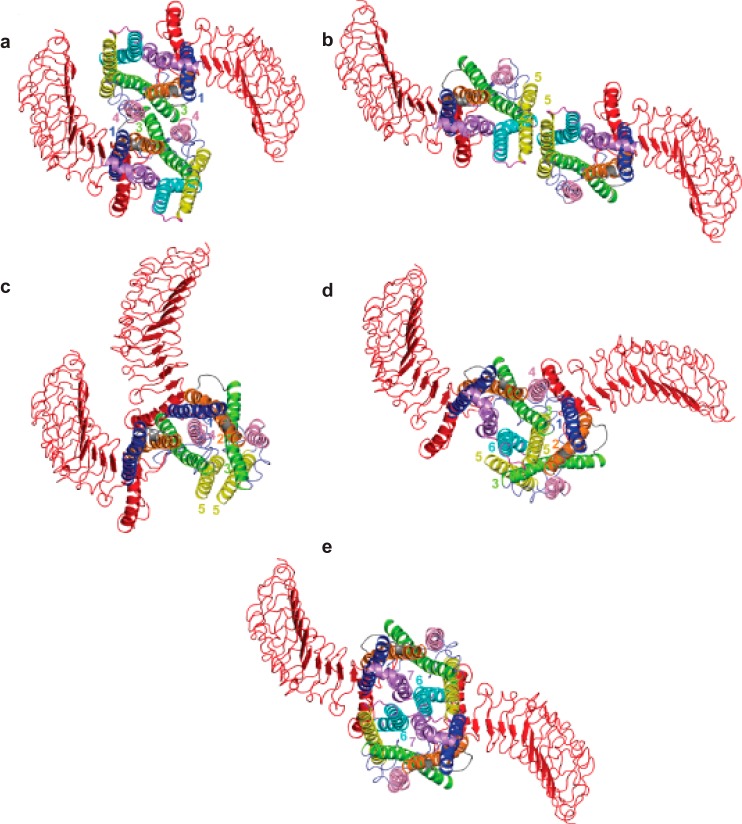
**Multiple interfaces mediate LHR di/oligomeric formation.** Computational modeling demonstrating potential homo- and heterodimer interfaces, with interfaces in H4-H1 and H3 (*a*) and H5 contacts (*b*), characteristic of and possibly through homomeric LHR^B−^ and LHR^S−^ or heteromeric LHR^B−^/LHR^S−^ interacting pairs. *c,* interfaces observed in H4, H5-H1, H3, and H5 are unique to the LHR^S−^ homomeric pair. *d,* interfaces observed in H4, H5, H6-H1, H2, H3, and H5 are unique to the heteromeric LHR^B−^/LHR^S−^ pair, and *e,* interface H6-H7 is unique to the homomeric LHR^B−^ interaction. The interfaces are shown from the intracellular side, perpendicular to the membrane plane. The receptor is divided into different colors as follows: ECD, *red*; TM helices: *1*, *blue*; *2*, *orange*; *3*, *green*; *4*, *pink*; *5*, *yellow*; *6*, *cyan*; and *7*, *purple* with helix 8 also represented in *purple*. Intracellular and extracellular loops (*IL* and *EL*, respectively) are depicted as follows: *1*, *slate*; *2*, *gray*; and *3*, *magenta*. Helices participating in the interface are labeled by the corresponding helix number.

To predict possible interfaces between LHR^B−^/LHR^S−^ oligomers, docking simulations were also carried out on dimers. Fig. 9 presents the most reliable self- and intermolecular cooperating dimer architectures that importantly also recurred in oligomeric complexes. Certain helix contact signatures were observed in all three possible pairings (self-associating LHR^B−^, LHR^S−^ dimers, and LHR^B−^/LHR^S−^ dimers), namely H4–H1, H3 (Fig. 9*a*), or H5 contacts (Fig. 9*b*). However, H4, H5-H1, H3, and H5 (Fig. 9*c*) was unique to the self-associating LHR^S−^ homomers. The more complex H4, H5, H6-H1, H2, H3, and H5 (Fig. 9*d*) contacts were unique to the intermolecular cooperating LHR^B−^/LHR^S−^ heteromers and H6-H7 contacts unique to the LHR^B−^ homomer (Fig. 9*e*). The H5-H5 interface, possible in both self-associating and intermolecular cooperating LHR^B−^/LHR^S−^ heteromers (Fig. 9*b*), and the H6-H7 interface unique to the LHR^B−^ homomers (Fig. 9*e*) permitted the largest separation between the two N termini. Inter-ECD contacts were rare and involved the ECD hinge region (although incomplete in our model due to current limitations of FSHR ECD crystal structures). Interestingly, despite deletion of H6 and H7 in LHR^S−^ that would limit the number of possible interfaces in the self-associating LHR^S−^ homodimer, this deletion increased the number of possible interacting interfaces in the LHR^B−^/LHR^S−^ heteromers (Fig. 9, *a, b,* and *d*). Collectively, the predicted and PD-PALM-visualized LHR^B−^/LHR^S^ di/oligomers are comprised of diverse and complex combinations of helix interfaces, with multiple possible inter-protomer interfaces.

## DISCUSSION

GPCR dimerization and oligomerization has been described nearly 2 decades ago (48), contributing to the recent paradigm shift in GPCR signaling, of a simplistic, archetypal view involving single receptors activating specific heterotrimeric G proteins at the cell surface to one of an increasing complex receptor signaling system. However, how this complexity generates functional pleiotropy in GPCR signaling, and our understanding of the contributions of oligomerization in this process, is poorly understood. One significant reason has been the lack of approaches to visualize individual GPCRs in a nondiffraction limited manner. Super-resolution imaging overcomes these limitations, providing a means to localize molecules at low nanometer resolution. Here, we employ dual-color super-resolution imaging, PD-PALM, to address how individual receptor protomers with defined activities operate within a GPCR di/oligomer to regulate ligand-induced responses. We propose that altering the functional role of individual protomers within an oligomer may provide an unprecedented mechanism to fine-tune receptor signaling.

The advantage of localization microscopy techniques such as PD-PALM is the high spatial resolution achieved (∼8 nm in the present study). Class A GPCRs are ∼6 nm (49), around 30-fold smaller than the diffraction limit of conventional microscopy. Therefore, PD-PALM resolves individual receptors in distinct dimeric and oligomeric complexes with differing protomer amounts, enabling quantification of LHR in monomers, dimers, and oligomers that range from trimers to higher order oligomers containing greater than nine receptors. To date, resonance energy transfer approaches, such as TR-FRET, and TIRF-M imaging, have been used to study GPCR di/oligomerization at the plasma membrane (15–17, 50). Although each of these techniques has its advantages, discrepancies still exist whether GPCRs form dimers or oligomers, even for the well studied β_2_-adrenergic receptor (23, 51–54). These differences may, at least in part, be due to resolution limits of the techniques employed (16, 50). For the higher order oligomers, *i.e.* >9 molecules, we cannot completely rule out the possibility of imaging receptors in defined membrane microdomains, although ligand-independent internalization was not observed for either WT or the mutant receptors (10). However, monomers, dimers, and lower order oligomers (<5 receptors) comprised >80% of the forms detected, even with PD-PALM enabling localization of labeled molecules at higher densities (∼200 molecules/μm^2^) than other techniques (1–2 molecules/μm^2^) (16, 55). As we could not detect a change in either the percentage of associated molecules or the relative proportions of dimers and oligomers following ligand treatment may suggest that the majority of these forms are not in membrane domains or clusters. However, ligand-induced changes in LHR complexes may be highly dynamic and possibly not be detectable via PD-PALM on cells that are fixed following ligand treatment. Importantly, the trimer and tetramer geometries imaged by PD-PALM showed high alignment with the structurally predicted models, despite the PD-PALM images integrating the PD-conjugated antibody. This suggests that the combination of these techniques provides a powerful approach to study individual receptor protomers participating in oligomerization in intact cells.

The current model of LHR intermolecular cooperation is based on formation of a dimer between the two nonfunctional mutant receptors, LHR^B−^ and LHR^S−^ (8, 9, 42). However, we observed both preformed dimers and oligomers, with the latter population being more prevalent. Single expression of intermolecular cooperating mutant receptors showed comparable numbers of monomers, dimers, and oligomers to WT LHR, suggesting that the preference for the co-expressed mutant receptors to form hetero-oligomers was due to a preferential association between LHR^B−^ and LHR^S^, rather than the result of structural changes the mutations introduced. This was supported by the structural modeling, which showed larger and more numerous interfaces operating in hetero-oligomers. Whether the structural requirements of the di/oligomer interfaces are the same interfaces involved in intermolecular cooperation, however, remains unclear but are likely to be distinct. Functional studies to identify the minimal functional unit to observe intermolecular cooperation have been conflicting, with one study indicating that is the ECD of LHR, anchored by CD8, to activate a LHR^B−^ (42), although other studies have observed that H1 is also required (43). Yet previous BRET studies to identify dimer/oligomer interfaces of FSHR demonstrated the involvement of both the ECD and transmembrane (TM) bundle (56). More recent reports have demonstrated that the transmission of conformational changes upon glycoprotein hormone binding occurs from the hormone-bound ECD of one protomer to the other protomer via the TM bundles, as opposed to direct communication of the ECDs (57). Indeed, our PD-PALM and structural modeling data support this idea and additionally suggest that multiple LHR helices can be involved in di/oligomer formation. Crystal structures reporting oligomeric formation of the β_1_-adrenergic receptor and the μ-opioid receptor indicate organization into a tetramer of a single specific arrangement, comprised of either linear dimers or parallel dimers and two distinct helix interfaces involving TM1, TM2, and either TM4–5 or TM5–6, depending on the receptor (58, 59). By combining super-resolution spatial analysis data with structural modeling, we observe similar receptor arrangements and helix associations, but we have also unveiled an unprecedented additional complexity and diversity in potential structural associations or assemblies that GPCR oligomers can undergo. Such increased complexity may be a reflection of capturing multiple oligomeric forms in intact cells. The potential increase in possible inter-protomer interfaces in the oligomeric complexes undergoing functional complementation may, at least in part, underlie the preferential formation of LHR^B−^/LHR^S−^ dimers and oligomers over self-associating homo-di/oligomers.

Although we could not detect ligand-induced changes in the total numbers of pre-existing di/oligomers, we did observe that intermolecular cooperation is sufficient to mediate full hCG- but not LH-mediated Gα_q/11_ responses. The reduced levels of LH-dependent Gα_q/11_ signaling may not be dictated by composition of the LHR^B−^/LHR^S−^ oligomer but rather the directionality of how LH activates individual protomers and transmits signals to neighboring protomers, as suggested by the differences observed between hCG and LH-dependent Gα_q_ BRET associations. For full LH-induced activation of Gα_q/11_ signaling, it is possible that the directionality of receptor activation may be via individual receptor protomers within a complex rather than activation of neighboring protomers, or even functional receptor WT monomers. However, how these ligands activate individual receptors remains to be determined.

The complexity and diversity in LHR oligomer formation, composition, and helix interfaces identified in this study may provide a mechanism for dynamic rearrangement of protomers, facilitating a highly exquisite mode of modulating signal specificity and/or strength. Such observations provide evidence for current conceptual models where GPCR oligomerization is a platform for diversifying/amplifying signal responses (1, 48). The composition of LHR^B−^/LHR^S−^ oligomers may regulate the amplitude of signal response, where increasing the total proportion of surface LHR^S−^ positively regulates the signal amplitude by increasing the number of LHR^B−^ participating in oligomers. By biasing the oligomeric complex toward “signaling-able” (LHR^B−^) protomers, it may provide a platform for further G protein couplings and consequently increased signal output. Our structural analysis suggests that enrichment of LHR^B−^ within LHR^B−^/LHR^S−^ oligomers permits the association of two trimeric G proteins via the H5 or H4-H1, H3 interfaces. In contrast, cells favoring the formation of 1B^−^:3S^−^ exhibited the lowest ligand-induced signal responses, perhaps due to the ability of such oligomers to accommodate one G protein while binding multiple hormone molecules. As glycoprotein hormone receptors display strong negative cooperativity at physiological hormone concentrations with a single agonist molecule binding to a receptor dimer (57), the number of G protein molecules associated in an oligomer is likely to be the limiting factor in regulating signal sensitivity.

In summary, by exploiting receptors that only signal via intermolecular cooperation, we have demonstrated that asymmetric GPCR oligomers can regulate the signaling profile of a receptor. Such a mechanism of receptor regulation, via diverse molecular arrangements of individual receptor mutant protomers with defined functions, provides a system to acutely fine-tune the specificity and signaling output, which may be critical for differential modulation of cellular responses in physiological or pathological contexts. Whether distinct complexes could be exploited in the development of new compounds remains to be determined, yet studying the molecular makeup via super-resolution techniques with computational structural modeling enables single molecule detection of the complete GPCR cell surface landscape with high definition, providing an approach to potentially design novel compounds with greater efficacy, selectivity, and/or specificity.

## Footnotes

3 The abbreviations used are:

GPCR: G protein-coupled receptor
BRET: bioluminescence resonance energy transfer
cre-luc: cyclic AMP-response element-luciferase
ECD: extracellular domain
FSHR: follicle-stimulating hormone receptor
H: helix
hCG: human chorionic gonadotropin
HTRF: homogenous time-resolved fluorescence
IP_1_: myo-inositol 1-phosphate
LH: luteinizing hormone
LHR: luteinizing hormone receptor
LHR^B−^: binding-deficient LHR mutant
LHR^S−^: signaling-deficient LHR mutant
PALM: photoactivated localization microscopy
PD-PALM: photoactivatable dye-photoactivated localization microscopy
TIRF-M: total internal reflection fluorescence microscopy
TM: transmembrane
ANOVA: analysis of variance
PD: photoactivatable dye.
